# Potential Roles of *Escherichia coli* in IgA Nephropathy: Evidence, Mechanistic Hypotheses and Microbiota‐Targeted Strategies

**DOI:** 10.1111/1751-7915.70455

**Published:** 2026-10-12

**Authors:** Yuxin Liu, Xiaoxuan Ning, Yichen Liu, Zixian Yu, Jie Liu, Xiaoqin Xu, Jin Zhao, Shiren Sun

**Affiliations:** ^1^ Department of Nephrology Xijing Hospital, The Fourth Military Medical University Xi'an Shaanxi China; ^2^ The Graduate School of Xi'an Medical University Xi'an Shaanxi China; ^3^ Department of Geriatrics Xijing Hospital, The Fourth Military Medical University Xi'an Shaanxi China; ^4^ Nanjing Medical University Nanjing Jiangsu China; ^5^ Yan'an University Yan'an Shaanxi China; ^6^ Shaanxi Provincial Clinical Research Center for Kidney Diseases Xi'an Shaanxi China

**Keywords:** *Escherichia coli*, gut‐kidney axis, IgA nephropathy, metabolic regulation, microbiota intervention, mucosal immunity

## Abstract

IgA nephropathy (IgAN), the most common primary glomerular disease, involves mucosa–kidney axis dysregulation, with gut dysbiosis increasingly implicated in its pathogenesis. Certain *Escherichia coli* ( *E. coli* ) strains harbour virulence‐associated features that promote intestinal colonization, barrier disruption and immune activation. Recent microbiome and metagenomic studies have reported an enrichment of *E. coli* in patients with IgAN, suggesting a potential link to the mucosa–kidney axis. Nevertheless, *E. coli* may contribute to IgAN through two major pathways. First, sustained LPS exposure may induce aberrant production of galactose‐deficient IgA1 (Gd‐IgA1) by stimulating B cells and activate the NLR family pyrin domain containing 3 (NLRP3) inflammasome in macrophages, thereby amplifying systemic and renal inflammation. Second, metabolic reprogramming is associated with increased circulating sphingosine‐1‐phosphate (S1P) and altered production of indole and trimethylamine (TMA); S1P may promote mesangial proliferation and fibrosis, whereas indole‐ and TMA‐related pathways may contribute to tubulointerstitial injury and chronic kidney disease (CKD) progression. Beyond conventional gut microbiota‐targeted interventions, emerging precision therapeutic strategies offer promising new directions for the etiological treatment of IgAN. This review summarizes *E. coli* virulence factors, pathogenesis, and host, environmental, and ecological factors driving its gut expansion in IgAN patients and outlines corresponding therapeutic strategies.

## Graphical abstract

Schematic diagram of the core pathogenic pathway by which *E. coli* drives IgAN.E. coli expansion in IgAN is driven by host, environmental and ecological factors, and promotes disease progression via two core pathways: LPS‐induced immune activation and pyroptosis, and metabolite‐mediated remote regulation, leading to Gd‐IgA1 overproduction, mesangial deposition, renal inflammation and fibrosis.

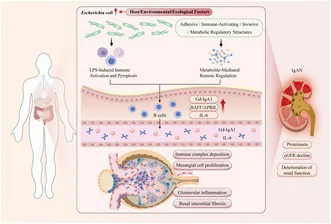

## 1 Introduction

IgA nephropathy is the most common primary glomerular disease worldwide, with substantial geographic variation in its reported incidence (Lee et al. 2023; Roberts et al. 2024). Population‐based annual incidence has been reported to be approximately 39–45 cases per million population in Japan, 25–31 in France, and 9.9 in the United Kingdom (Lee et al. 2023). Comparable population‐based incidence data are less consistently reported for China; however, IgAN accounts for approximately 30%–40% of primary glomerular diseases in Chinese biopsy‐based cohorts (Chen et al. 2023; Xu et al. 2025). These geographic differences in reported disease frequency may reflect genetic susceptibility as well as differences in population screening and kidney biopsy practices. The clinical manifestations of IgAN range from asymptomatic hematuria to rapidly progressive glomerulonephritis (Remuzzi et al. 2006; Wang et al. 2014; Ebbestad et al. 2022). Diagnosis is primarily confirmed by kidney biopsy showing dominant mesangial IgA deposition (Berger and Hinglais 1968; Rovin et al. 2021). Accumulating evidence links pathogenic Gd‐IgA1 to dysregulated mucosal immunity (Coppo 2018; Roberts et al. 2024; Stoneman et al. 2026; Wu et al. 2026). Loss of immune tolerance to commensal microbiota may activate B cells and promote aberrant Gd‐IgA1 production, contributing to glomerular deposition and renal injury. Gut microbiota dysbiosis may provide persistent mucosal immune stimulation and contribute to mucosal inflammation (Sekirov et al. 2010; Belkaid and Hand 2014; Marchesi et al. 2016; Sterling et al. 2022; Di Giorgio et al. 2023; Shen et al. 2025).

Several independent cohort studies have reported enrichment of the *Escherichia‐Shigella* group in the gut microbiota of patients with IgAN compared with healthy individuals and/or disease controls (Dong et al. 2020; Hu et al. 2020; Zhong et al. 2020; Zhao et al. 2022; Dong, Yan, et al. 2025; Lopes et al. 2025). More recently, metagenomic sequencing has identified *E. coli* as a species‐level taxon enriched in patients with IgAN (Deng et al. 2024). Importantly, *E. coli* is also a normal member of the human gut microbiota, with commensal strains contributing to colonization resistance through competition for nutrients and ecological niches (Conway and Cohen 2015). Given its substantial strain‐level diversity, enrichment of *E. coli* in IgAN should be interpreted in the context of gut dysbiosis rather than as evidence that all strains contribute to disease. The specific *E. coli* strains or functional characteristics associated with IgAN remain unknown (Deng et al. 2024). Meanwhile, recent studies have found that gut *E. coli* can participate in the pathogenesis of CKD through multiple mechanisms, and these findings further support investigation into its potential role in the gut–kidney axis in IgAN (Coppo 2018; Zhu et al. 2024).

This review focuses on *E. coli* as a recurrently reported bacterial taxon associated with IgAN, summarizes the clinical evidence linking it to IgAN, examines the potential mechanisms by which *E. coli* ‐associated dysbiosis may contribute to Gd‐IgA1 production and kidney injury, and discusses microbiota‐targeted therapeutic strategies and future research directions (Figure 1).

**FIGURE 1:**
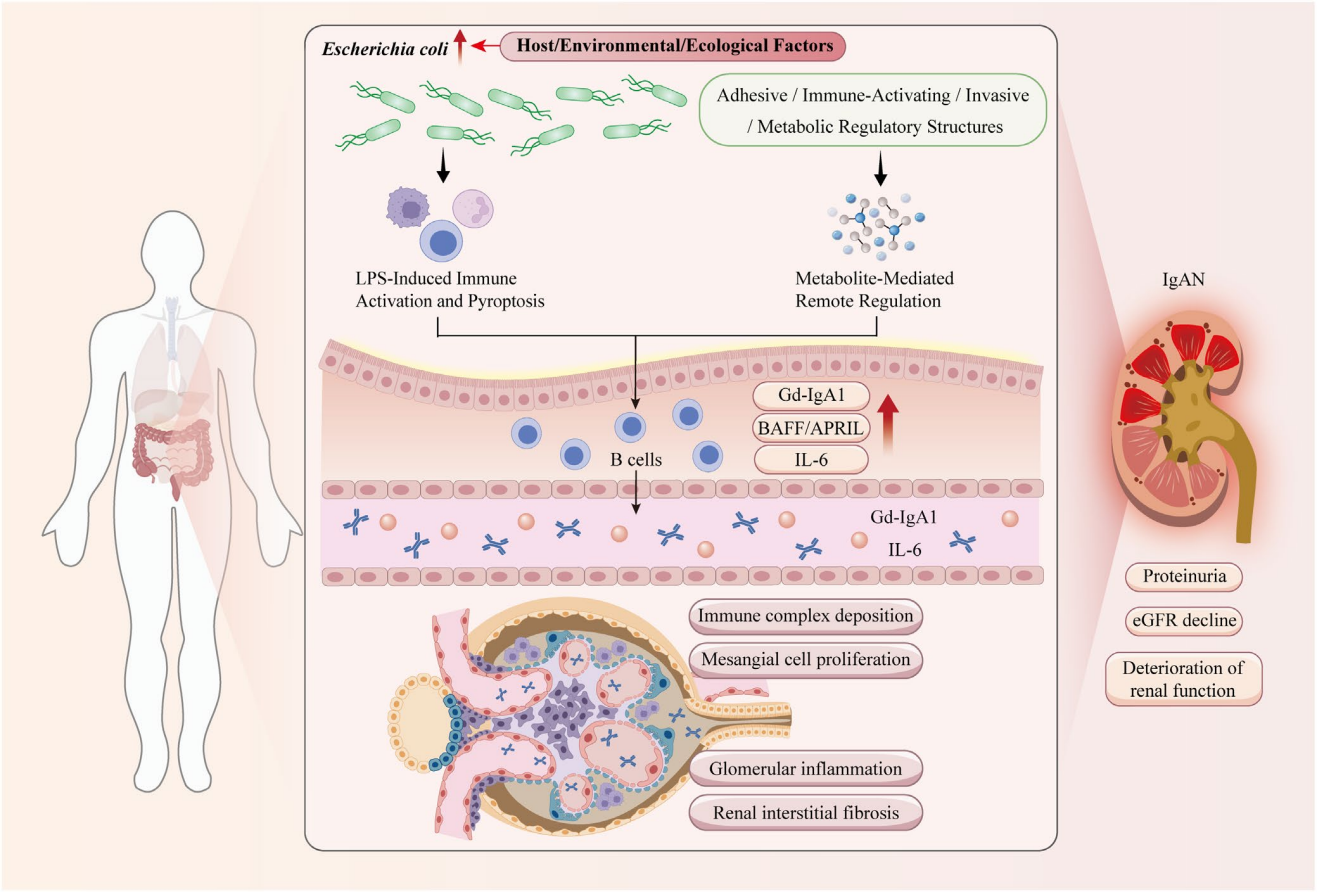
Schematic diagram of the proposed mechanistic framework linking *E. coli* ‐associated gut dysbiosis to IgAN. *E. coli* enrichment in IgAN may be influenced by host, environmental, and ecological factors and may contribute to disease‐related processes through LPS‐associated immune activation and pyroptosis and metabolite‐mediated remote regulation.

## 2 Clinical Associations Between *E. coli* and IgAN

### 2.1 Key Clinical Cohort Studies

Several clinical cohort studies have consistently reported an increased abundance of the *Escherichia‐Shigella* group in patients with IgAN, although study design, control populations, and clinical associations vary among cohorts. Dong et al. (2020), Hu et al. (2020), Zhong et al. (2020), and Zhao et al. (2022) reported enrichment of *Escherichia‐Shigella* in IgAN using 16S rRNA sequencing. Several of these studies further identified associations with clinical or immunological indices, including proteinuria, hematuria, eGFR, uACR, and treatment response. Zhu et al. (2024) additionally linked *Escherichia‐Shigella* enrichment with circulating Gd‐IgA1, while Dong, Zhang, et al. (2025) reported greater abundance in patients with an unfavourable treatment response.

Importantly, these 16S‐based findings generally resolve the signal at the *Escherichia‐Shigella* taxonomic level and therefore cannot establish that the observed differences are attributable specifically to *E. coli* or to particular strains. Deng et al. (2024) subsequently used metagenomic sequencing and identified *E. coli* as a species‐level taxon enriched in IgAN, providing more direct species‐level evidence. Nevertheless, the available studies remain predominantly cross‐sectional, and strain‐resolved analyses are required to determine whether specific *E. coli* lineages contribute disproportionately to disease‐associated dysbiosis (Table 1).

**TABLE 1:** Summary of core clinical cohort studies investigating the association between *E. coli* and IgAN.

Journal	Sample size (IGAN/control)	Detection technology	Key findings ( *E. coli* )	Control group type	References
J Am Soc Nephrol	127/127	16S rRNA (V3‐V4)	The *Escherichia‐Shigella* group (with a constituent ratio of 10.0% in patients vs. 1.0% in controls) is enriched in patients with IgAN, and its abundance is positively correlated with serum creatinine, proteinuria, and the severity of Oxford pathological classification, while negatively correlated with albumin and eGFR.	Healthy volunteers + Patients with membranous nephropathy	Zhao et al. (2022)
Frontiers in Cellular and Infection Microbiology	44/30/40	16S rRNA (V3‐V4)	The *Escherichia‐Shigella* group (with a relative abundance of 5.67% to 1.04%) was enriched in IgAN patients, and its abundance was negatively correlated with proteinuria.	Healthy volunteers + Patients with membranous nephropathy	Dong et al. (2020)
Nephrol Dial Transplant	48/31/22	16S rRNA	The *Escherichia‐Shigella* group was enriched in untreated IgAN patients, and the degree of enrichment was positively correlated with the level of Gd‐IgA1.	Healthy volunteers + Patients with membranous nephropathy Patients with IgAN who have received treatment	Zhu et al. (2024)
International Immunopharmacology	52/25	16S rRNA (V3‐V4)	The *Escherichia‐Shigella* group is enriched in patients with IgAN, and its abundance is positively correlated with hematuria and proteinuria.	Healthy volunteers	Zhong et al. (2020)
Renal Failure	55 (no control group)	16S rRNA (V3‐V4)	The *Escherichia‐Shigella* group is the most significant differential genus in the gut microbiota of non‐responsive patients. This genus is the main producer of endotoxins and LPS.	Non‐responsive IgAN patients	Dong, Yan, et al. (2025)
BMC Nephrology	17/18	16S rRNA (V3–V4)	The *Escherichia‐Shigella* group was enriched in patients with IgAN, and its abundance was negatively correlated with eGFR and positively correlated with uACR.	Healthy volunteers	Hu et al. (2020)
Renal Failure	40/20	Metagenomic sequencing	The characteristic bacterium was identified at the species level as *E. coli* (14.01% in the IgAN group vs. 5.54% in the healthy control group, *p* = 0.0288) and was positively correlated with IgAN (*r* = 0.333, *p* = 0.009).	Healthy volunteers	Deng et al. (2024)

*Note:* The aforementioned studies reveal three key points of consensus: ① Consistency of findings: The relative abundance of the intestinal *E. coli* is significantly higher in patients with IgAN compared to control populations, with enrichment ratios ranging from an increase of 1.04% to 5.67% and from 1.0% to 10.0%, all demonstrating statistical significance. ② Disease specificity: enrichment is specific to IgAN (not observed in membranous nephropathy controls). ③ Early pathological association: enrichment occurs in newly diagnosed patients, correlates with Gd‐IgA1, and is linked to non‐responsive phenotype and endotoxin production. ④ Metagenomics identifies *E. coli* at the species level as a characteristic bacterium in IgAN, with abundance positively correlated with the disease.

### 2.2 Strength of Evidence and Alternative Interpretations

Although several clinical studies have reported enrichment of *E. coli* in patients with IgAN, the current evidence should be interpreted cautiously. Most available studies are cross‐sectional and based on 16S rRNA sequencing, which limits species‐ and strain‐level resolution and cannot reliably distinguish commensal *E. coli* from potentially pathogenic lineages. Therefore, the observed enrichment of *E. coli* should be regarded as an association rather than direct evidence of causality.

Alternative explanations for the observed *E. coli* enrichment should also be considered. Diet and medication exposure may act as upstream influences on the gut microbiota, whereas broader dysbiosis and depletion of butyrate‐producing bacteria may represent associated ecological changes. Impaired intestinal barrier function and disease‐related inflammation may be both consequences and drivers of microbial alterations. These factors may also confound the associations observed in predominantly cross‐sectional studies. In addition, several proposed mechanisms, including LPS‐mediated immune activation, virulence factor–related barrier injury, and metabolite‐mediated renal effects, are biologically plausible but remain incompletely validated in human IgAN. Thus, throughout this review, we distinguish established clinical observations from mechanistic hypotheses and emerging evidence requiring further validation.

## 3 Biological Features and Drivers of Intestinal Enrichment of *E. coli* in IgAN

### 3.1 Potentially Relevant Virulence‐Associated Features of *E. coli*

Although it remains unknown whether *E. coli* strains enriched in IgAN represent pathogenic lineages, several features characterized in specific *E. coli* strains provide plausible mechanisms for intestinal colonization, immune activation, and barrier disruption.

#### 3.1.1 Adhesion‐ and Barrier‐Disrupting Structures

The core adhesive structures of *E. coli* are fimbriae (e.g., Type 1 fimbriae, P fimbriae). The adhesins located at their tips, such as the FimH protein of Type 1 fimbriae, specifically recognize and bind to mannose receptors on the surface of intestinal epithelial cells, forming a stable attachment (Krogfelt et al. 1990). This adhesion enables *E. coli* to resist clearance by intestinal peristalsis, thereby establishing long‐term colonization (Miller et al. 2006; Bachmann et al. 2025). Additionally, P fimbriae and Afa/Dr. adhesins expressed by pathogenic *E. coli* can recognize receptors such as CD55 and CEACAM on intestinal epithelial cells, facilitating robust adhesion and invasion (Korotkova et al. 2006).

In addition to promoting adhesion and intestinal persistence, certain pathogenic *E. coli* lineages, particularly enteropathogenic *E. coli* (EPEC), employ a Type III secretion system (T3SS) encoded by the locus of enterocyte effacement (LEE) to translocate effector proteins into host epithelial cells (Elliott et al. 2001). Among these, EspF compromises intestinal barrier integrity by downregulating the expression of tight junction proteins, promoting their degradation, and interfering with the interaction between ZO‐1 and the actin cytoskeleton (Singh et al. 2018). EspG is identified as a key effector that disrupts microtubules and regulates vesicle trafficking, and it can synergize with EspI to affect intestinal absorption and barrier integrity (Clements et al. 2012). These adhesion‐ and barrier‐disrupting structures and mechanisms are summarized in Figure 2.

**FIGURE 2:**
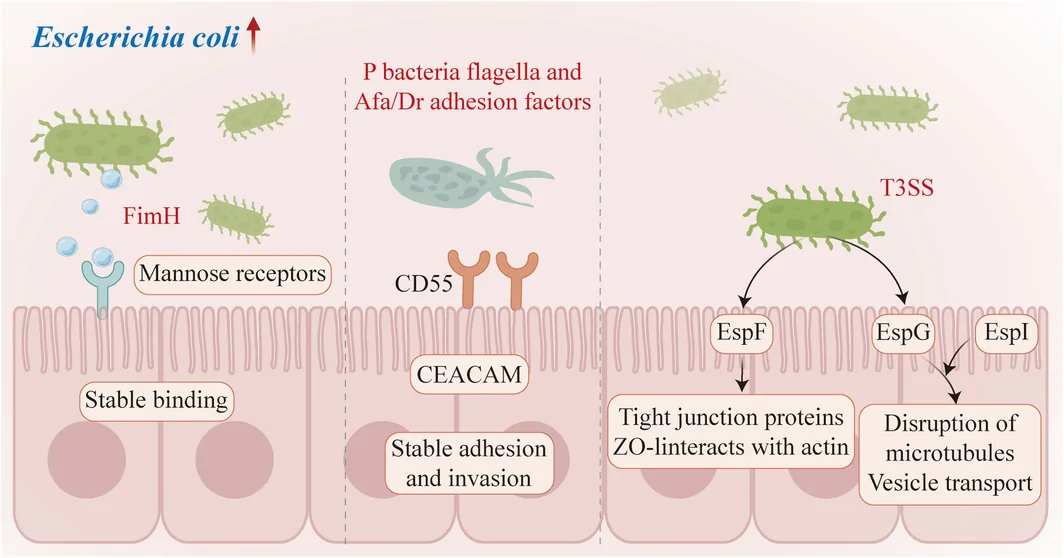
Virulence‐associated structures of *E. coli* involved in intestinal adhesion and barrier disruption. (A) Adhesion‐associated structures of *E. coli* . Type 1 fimbriae, P fimbriae, and Afa/Dr. adhesins mediate attachment to intestinal epithelial cells through interactions with specific host receptors. (B) Effector delivery mechanisms characterized in pathogenic *E. coli* . Enteropathogenic *E. coli* (EPEC) employs a LEE‐encoded type III secretion system (T3SS) to deliver effectors such as EspF and EspG, which disrupt tight junctions, microtubules, and vesicle trafficking. CD55, decay‐accelerating factor; CEACAM, carcinoembryonic antigen‐related cell adhesion molecule; EspF, *E. coli* secreted protein F; EspG, *E. coli* secreted protein G; EspI, *E. coli* secreted protein I; FimH, type 1 fimbrial adhesin FimH; LEE, locus of enterocyte effacement; T3SS, type III secretion system; ZO‐1, zonula occludens‐1.

#### 3.1.2 Immune‐Activating Structures

*E. coli* can release the immune‐activating molecules LPS and functional flagellin (Tominaga et al. 2001). LPS is a major component of the Gram‐negative bacterial cell wall and can be recognized by host immune cells as a pathogen‐associated molecular pattern (PAMP) (Koch et al. 2014). Flagellin, the structural subunit of bacterial flagella, is recognized by TLR5 and can trigger innate immune activation (Hayashi et al. 2001). Additionally, the *E. coli* adhesin FimH can bind to TLR4 on the surface of host dendritic cells (DCs), inducing them to secrete pro‐inflammatory cytokines such as IL‐6 and IL‐12, which in turn promote Th1/Th17 responses and thereby exacerbate inflammation (Zhang et al. 2023). Furthermore, *E. coli* continuously releases outer membrane vesicles, which upon uptake, trigger a robust innate immune response (Zhao et al. 2022). These immune‐activating structures and pathways are depicted in Figure 3.

**FIGURE 3:**
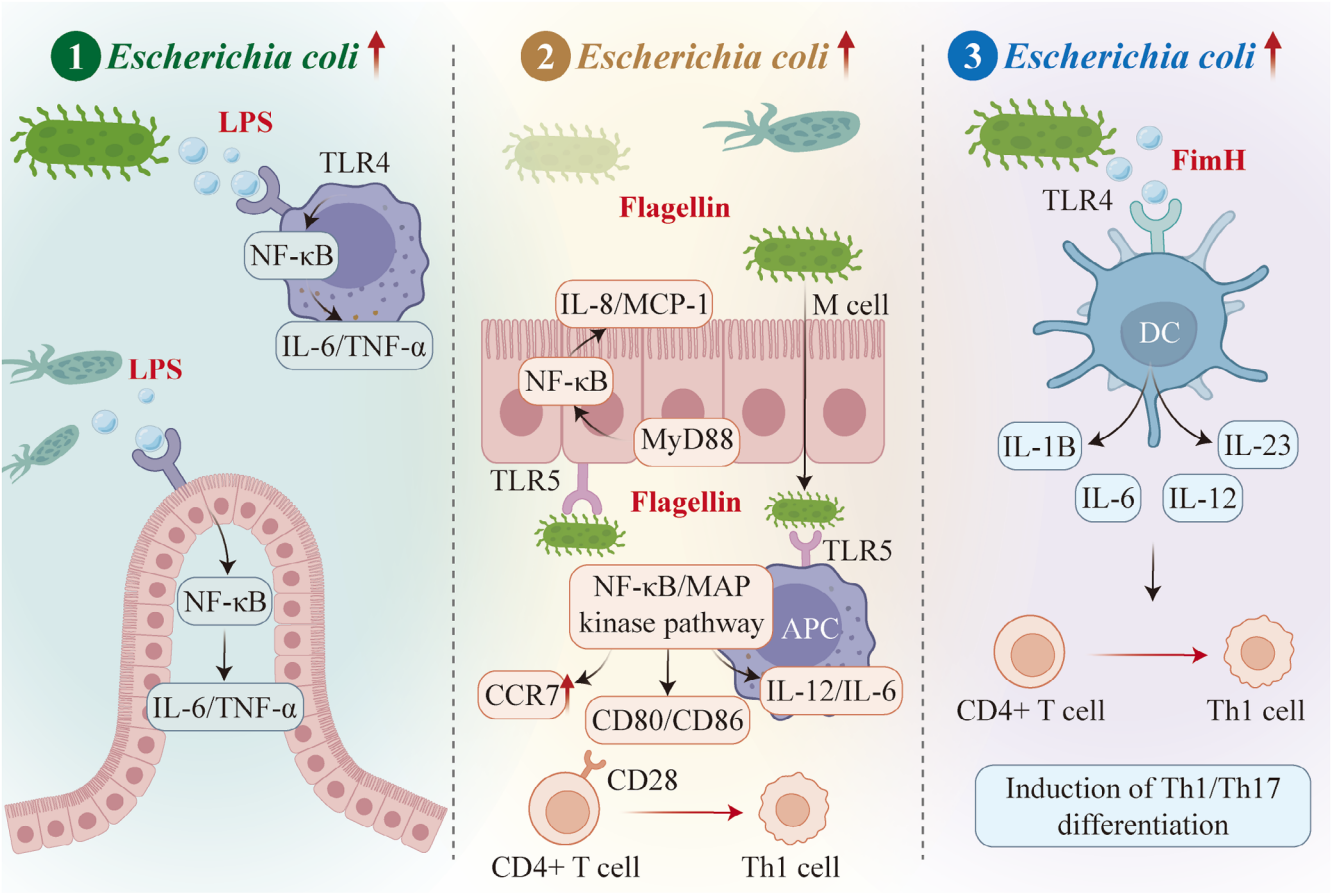
Schematic diagram of immune‐activating structures expressed by *E. coli* . *E. coli* can release LPS, which binds to TLR4 on immune and epithelial cells, activating the NF‐κB pathway and promoting pro‐inflammatory cytokine release (IL‐6, IL‐12). Flagellated *E. coli* strains produce flagellin, which activates TLR5 on dendritic cells, facilitating T and B cell proliferation. The FimH adhesin can also bind TLR4 on dendritic cells, inducing Th1/Th17 responses. APC, antigen‐presenting cell; CCR7, C‐C chemokine receptor type 7; CD28, cluster of differentiation 28; CD80/CD86, cluster of differentiation 80/86; DC, dendritic cell; IL‐12, interleukin‐12; IL‐1β, interleukin‐1 beta; IL‐23, interleukin‐23; IL‐6, interleukin‐6; IL‐8, interleukin‐8; M cell, microfold cell; MAP kinase, mitogen‐activated protein kinase; MCP‐1, monocyte chemoattractant protein‐1; MyD88, myeloid differentiation primary response 88; NF‐κB, nuclear factor kappa‐B; Th1 cell, T helper type 1 cell; TLR4, Toll‐like receptor 4; TLR5, Toll‐like receptor 5; TNF‐α, tumour necrosis factor‐alpha.

#### 3.1.3 Metabolic Regulatory Structures

Recent experimental evidence has linked *E. coli* ‐associated gut dysbiosis to altered sphingolipid metabolism and increased circulating S1P levels in IgAN models. Elevated circulating S1P acts on renal mesangial cells by activating the C‐C motif chemokine ligand 2 (CCL2)‐MET proto‐oncogene (MET)‐focal adhesion kinase (FAK) signalling axis, driving mesangial cell proliferation, inhibiting apoptosis, and promoting IgA deposition, thereby accelerating the progression of IgAN (Tang et al. 2025). This metabolite‐mediated regulatory mechanism is shown in Figure 4.

**FIGURE 4:**
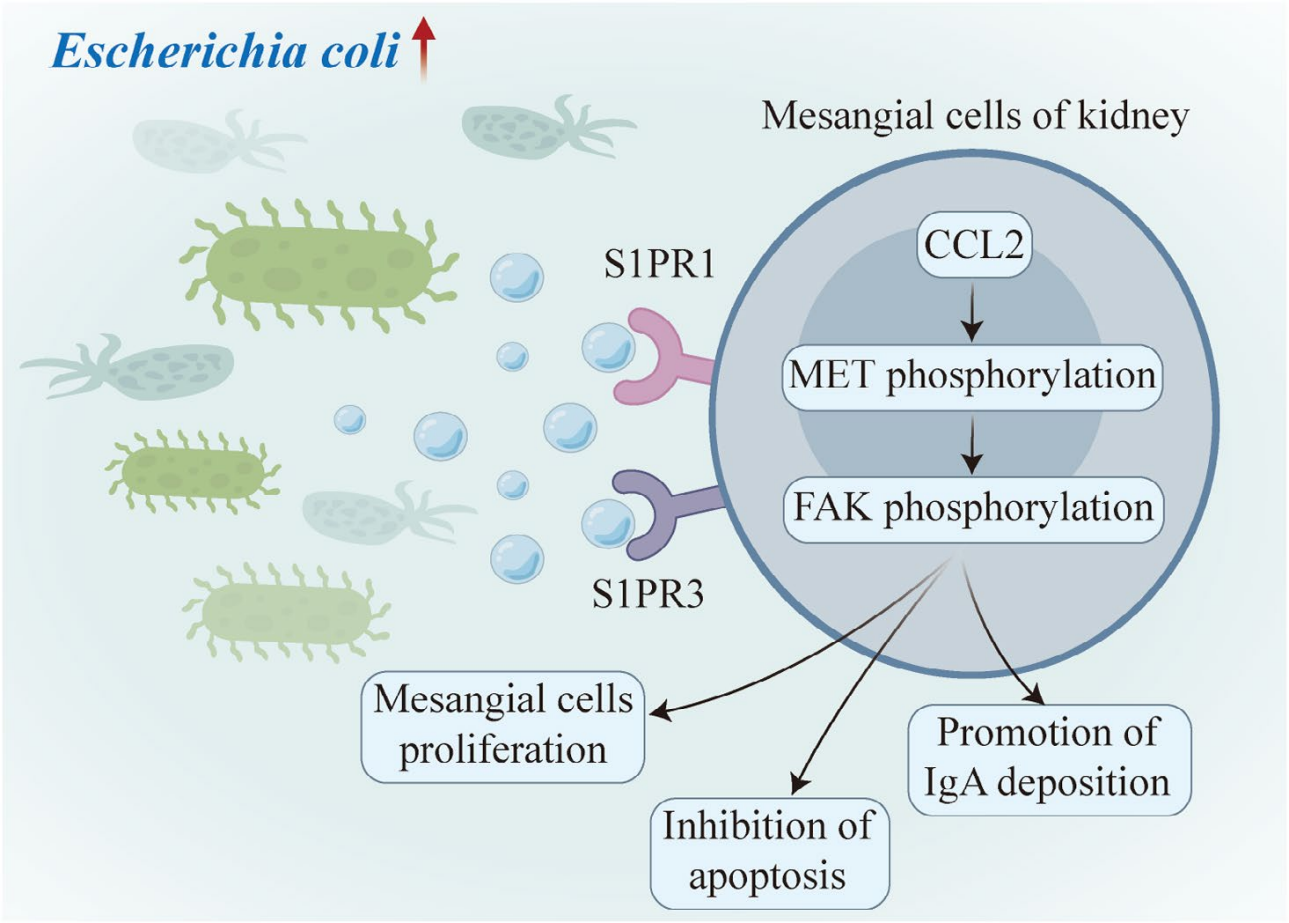
Schematic diagram depicting the metabolic regulatory structures of *E. coli* . Increased circulating S1P associated with gut *E. coli* dysbiosis can act on glomerular mesangial cells via S1PR1/S1PR3 receptors, activating the CCL2‐MET‐FAK signalling axis, thereby promoting mesangial cell proliferation and facilitating IgA deposition, which ultimately accelerates the progression of IgAN. CCL2, C‐C motif chemokine ligand 2; FAK, focal adhesion kinase.MET, MET proto‐oncogene, receptor tyrosine kinase; S1PR1, sphingosine‐1‐phosphate receptor 1; S1PR3, sphingosine‐1‐phosphate receptor 3.

### 3.2 Drivers of Intestinal Enrichment of *E. coli* in IgAN

The reported enrichment of *E. coli* in the gut microbiota of patients with IgAN may reflect the combined influence of host factors, alterations in the intestinal microenvironment, and inter‐microbial interactions.

#### 3.2.1 Host Factors

Host genetic factors contribute to *E. coli* expansion in IgAN. A genome‐wide association study identified that loss‐of‐function variants in *SIPA1L3* and *AL392086.3* correlate with increased gut Proteobacteria (including *E. coli* ) abundance (Wang et al. 2025). In addition, IgAN susceptibility genes involved in mucosal immunity may further weaken host constraints on *E. coli* colonization: copy number variations in *DEFA* impair the mucosal barrier, *CARD9* variants compromise antibacterial immunity, *TNFSF13* dysregulation enhances B cell responses to *E. coli* , and *LIF/OSM* variants alter the intestinal niche (Kiryluk et al. 2013; Zhang et al. 2017). Collectively, these genetic variants lead to aberrant mucosal immune responses that indirectly facilitate *E. coli* colonization and expansion in IgAN patients.

#### 3.2.2 Environmental Factors

Dietary patterns and intestinal metabolic remodelling in IgAN patients provide a selective growth advantage for *E. coli* (Zhao et al. 2022). Higher intake of fibre, vegetables, and fruits is associated with lower *E. coli* abundance (Zhang et al. 2020). Mechanistically, dietary fibre is fermented to butyrate (Louis and Flint 2017), which inhibits *E. coli* colonization and expansion via intracellular acidification, barrier enhancement, immune regulation, and maintenance of intestinal anaerobiosis (Bäumler et al. 2018; Zhan et al. 2022; Liu et al. 2024). In addition, a gluten‐free diet increases *E. coli* and Enterobacteriaceae counts (Collins et al. 2009; Sanz 2010). Environmental chemical exposures may also influence *E. coli* expansion by altering the intestinal ecological environment. In vitro faecal fermentation experiments showed that exposure to dishwashing detergent or cinnamaldehyde increased *E. coli* growth while reducing butyrate production and several butyrate‐producing bacterial populations (Gerasimidis et al. 2019). These differential effects on microbial populations and fermentation suggest that certain chemical exposures may create ecological conditions favouring *E. coli* expansion, although the underlying mechanism has not been directly established. Consistent with a possible role of environmental exposures in IgAN‐associated microbial remodelling, predicted microbial pathways involved in xenobiotic biodegradation and metabolism have been reported to be enriched in patients with IgAN (Zhao et al. 2022); however, whether such exposures directly contribute to *E. coli* expansion in IgAN remains unknown.

#### 3.2.3 Ecological Factors

From an ecological perspective, the gut microbiota maintains a competitive equilibrium between fibre‐fermenting, butyrate‐producing taxa and opportunistic pathogens including Enterobacteriaceae. The Foundation Guild acidifies the intestinal lumen and suppresses pathogenic expansion, whereas the Pathobiont Guild carries abundant virulence and resistance genes (Wu et al. 2024). In IgAN patients, previous studies have reported reduced levels of butyrate‐producing bacteria, such as *Butyricicoccus* and *Eubacterium rectale* , as well as decreased lactate‐producing bacteria (*Lactobacillus* and *Bifidobacterium*) (Scott et al. 2022; Popova et al. 2025; Zhang, Han, et al. 2025). This depletion of the Foundation Guild collapses the ecological equilibrium, tilting the seesaw toward the Pathobiont Guild and thereby creating a niche permissive for *E. coli* proliferation (Wu et al. 2024). Mechanistically, butyrate deficiency compromises intestinal barrier integrity and increases permeability, promoting *E. coli* invasion, while reduced lactate supply further lowers butyrate levels (Hamed et al. 2023; Kalkan et al. 2025).

## 4 Mechanistic Links Between *E. coli* and IgAN

### 4.1 LPS‐Induced Immune Activation and Pyroptosis

In patients with IgAN, enrichment of *E. coli* in the gut may contribute to an increased intestinal LPS burden (Zhao et al. 2022; Gao et al. 2024; Zhu et al. 2024) (Figure 5). Concurrently, these patients often exhibit impaired intestinal barrier function (e.g., reduced expression of tight junction proteins), allowing LPS to enter the circulation (Zhou et al. 2020; Tang et al. 2023; Zhang, Ji, et al. 2025). The hexa‐acylated lipid A moiety of *E. coli* ‐derived LPS is a potent TLR4 agonist (Saha et al. 2022), and once in the bloodstream, LPS contributes to the initiation and progression of IgAN through two parallel pathways by stimulating TLR4.

**FIGURE 5:**
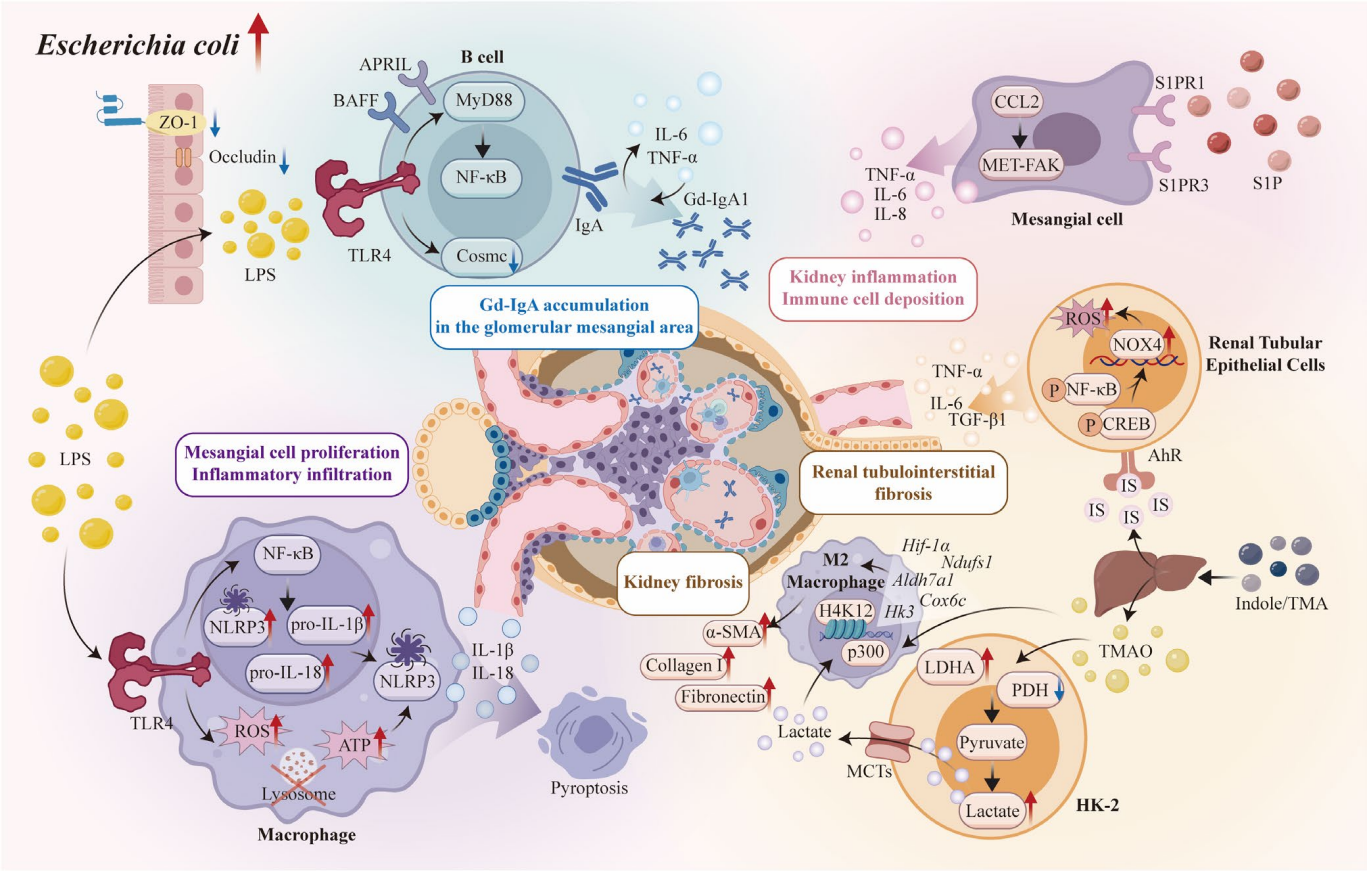
Proposed mechanistic pathways linking *E. coli* to IgAN. *E. coli* may contribute to IgAN‐related processes through two putative pathways: ① LPS‐induced immune activation and pyroptosis: *E. coli* , as a Gram‐negative bacterium enriched in IgAN‐associated gut dysbiosis, represents a potential contributor to the intestinal LPS burden. Increased intestinal LPS enters the circulation through an impaired intestinal barrier, activates TLR4/MyD88/NF‐κB signalling in B cells to promote Gd‐IgA1 production, and triggers NLRP3 inflammasome‐mediated pyroptosis in macrophages to amplify renal inflammation; ② metabolite‐mediated remote regulation: *E. coli* ‐associated alterations in S1P metabolism may increase circulating S1P, which activates the CCL2–MET–FAK axis in mesangial cells to promote mesangial proliferation, while indole‐ and TMA‐related pathways may represent additional mechanisms relevant to renal injury. FAK, focal adhesion kinase; MET, MET proto‐oncogene.

On one hand, as a ligand for TLR4, LPS activates the MyD88/NF‐κB signalling pathway in B cells. This activation produces two effects: (i) upregulation of B‐cell activating factors B‐cell activating factor (BAFF) and APRIL, which promotes B‐cell differentiation into IgA‐secreting plasma cells; and (ii) downregulation of the molecular chaperone Cosmc, leading to defective O‐glycosylation of IgA1 and the formation of Gd‐IgA1 (Hornef et al. 2003; Zhu et al. 2024; Fu et al. 2025). Concurrently, this pathway also enhances the release of pro‐inflammatory cytokines such as IL‐6 and TNF‐α, collectively driving the overproduction of Gd‐IgA1. These Gd‐IgA1 molecules eventually form immune complexes that deposit in the glomerular mesangium, triggering renal injury (Lim et al. 2011).

On the other hand, circulating LPS is also recognized by TLR4 on the surface of immune cells such as macrophages, activating the NF‐κB signalling pathway and upregulating the expression of NLRP3, pro‐IL‐1β, and pro‐IL‐18 (signal 1). Meanwhile, *E. coli* can induce a range of cellular stress responses—including LPS‐induced reactive oxygen species production, lysosomal damage, and ATP release from injured cells—providing a second signal that promotes the assembly of the NLRP3 inflammasome and activation of caspase‐1. This in turn cleaves gasdermin D (GSDMD), triggering pyroptosis, and facilitates the massive secretion of mature IL‐1β and IL‐18. These inflammatory cytokines enter the circulation and further exacerbate systemic and local renal inflammation, promoting mesangial cell proliferation, inflammatory cell infiltration, and renal interstitial fibrosis, thereby accelerating the progression of IgAN (Wu et al. 2022; Cheng et al. 2024).

Together, these findings support a proposed mechanistic framework in which increased LPS exposure may promote B‐cell–mediated Gd‐IgA1 production and macrophage‐mediated inflammatory responses. However, whether these processes form an integrated causal pathway in human IgAN remains to be directly demonstrated.

### 4.2 Metabolite‐Mediated Remote Regulation

Recent metabolomic studies have revealed that *E. coli* ‐associated metabolic alterations may provide an additional pathway linking gut dysbiosis to renal injury in IgAN.

In particular, *E. coli* ‐associated alterations in sphingolipid metabolism have been linked to increased circulating S1P levels. S1P is a bioactive sphingolipid that is naturally generated in mammalian cells through phosphorylation of sphingosine by sphingosine kinases (SphK1 and SphK2). Circulating S1P is mainly derived from host cells, particularly erythrocytes and endothelial cells, and regulates physiological processes including immune‐cell trafficking and vascular integrity (Maceyka and Spiegel 2014; Książek et al. 2015). Serum S1P levels are significantly elevated in IgAN patients compared to healthy controls and positively correlate with intestinal *E. coli* abundance. Experimental IgAN models further showed that *E. coli* ‐associated alterations were accompanied by increased circulating S1P levels (Schwalm et al. 2013, 2024; Tang et al. 2025; Zhang, Tang, et al. 2025). However, current evidence does not demonstrate that wild‐type *E. coli* directly produces S1P; rather, these findings suggest that *E. coli* ‐associated dysbiosis may influence host sphingolipid metabolism, although the underlying mechanism remains unclear.

Mechanistically, circulating S1P acts on S1PR1/S1PR3 receptors on glomerular mesangial cells, activating the CCL2‐MET‐FAK signalling axis. Activation of this signalling axis leads to three major pathological consequences: first, it drives abnormal mesangial cell proliferation, promoting glomerular mesangial expansion; second, it induces mesangial cells to secrete chemokines, recruiting immune cell infiltration and exacerbating local inflammation; and third, it upregulates the expression of IgA receptors on mesangial cells, enhancing the capture and retention of circulating Gd‐IgA1 (Zhang et al. 2018; Drexler et al. 2021; Tang et al. 2025). The S1P‐mediated remote regulatory pathway provides a potential link between *E. coli* ‐associated metabolic alterations and renal injury in IgAN and may represent a target for future therapeutic intervention. In addition to S1P, *E. coli* produces other metabolites that contribute to the progression of CKD, which may also accelerate the decline in renal function in patients with IgAN. For example, *E. coli* metabolizes dietary tryptophan into indole, which is further converted into indoxyl sulfate (IS) in the liver. IS activates the aryl hydrocarbon receptor (AhR) in renal tubular epithelial cells, inducing oxidative stress and the expression of pro‐inflammatory cytokines (e.g., TGF‐β1, MCP‐1), ultimately leading to tubulointerstitial fibrosis (Zhang, Tang, et al. 2025; Lee et al. 2026). Similarly, *E. coli* can metabolize dietary choline and carnitine to produce TMA, which is oxidized in the liver to trimethylamine N‐oxide (TMAO). Elevated TMAO levels are associated with CKD progression and cardiovascular risk (Tang and Hazen 2014; Kalniņš et al. 2015; Zhu et al. 2018; Zhou et al. 2022; Tang et al. 2026). Although direct evidence in IgAN is lacking, these metabolite‐driven pathways may represent additional mechanisms by which gut *E. coli* may contribute to renal injury and disease progression in IgAN.

## 5 Microbiota Dysbiosis as a Potential Therapeutic Target

Gut microbiota dysbiosis represents a potential therapeutic target in IgAN. Current microbiota‐targeted approaches include faecal microbiota transplantation (FMT), probiotics, dietary intervention, phage therapy, and engineered biological strategies; however, these strategies vary considerably in research maturity and level of clinical evidence. This section will review them systematically (Table 2).

**TABLE 2:** Potential microbiota‐targeted and precision therapeutic strategies relevant to IgA nephropathy.

Intervention strategy	Core mechanism	The impact on *E. coli*	Advantage	Limitations
Faecal microbiota transplantation (FMT)	Reconstitution of overall gut microbiota, modulation of gut metabolites and B‐cell immunity	Decreased abundance	Well tolerated; no severe adverse effects; reduces proteinuria	Small sample sizes; incomplete mechanistic understanding; requires large‐scale validation
Probiotics	Competitive exclusion of pathogens; production of SCFAs (e.g., butyrate) to enhance intestinal barrier function	Abundance decreased; LPS/endotoxin levels reduced	Good safety profile; suitable for long‐term adjunctive therapy	Strain‐specific effects; lack of standardized protocols; efficacy may vary
Glucocorticoids/immunosuppressants	Suppress mucosal immune activation, reduce intestinal inflammation, and indirectly limit pathogen expansion	Abundance significantly decreased (e.g., from 11.8% to 3.3% in responders)	Well‐established efficacy in controlling disease activity	Long‐term use may disrupt gut microbiota diversity; increased risk of infection and metabolic side effects
Dietary intervention (high fibre/gluten‐free)	Promote SCFA‐producing bacteria; enhance barrier function; reduce mucosal immune activation	Indirect inhibition of pathogen expansion	Non‐invasive, low‐cost; can be combined with other therapies	Lack of high‐quality evidence for efficacy as monotherapy; adherence may be challenging
Engineered IgA Protease	Directly cleaves Gd‐IgA1 molecules; Fc‐AK183 fusion protein eliminates deposited IgA and C3 in glomeruli	No direct impact (targets IgA rather than bacteria)	Directly addresses core pathological link; potential for rapidly progressive IgAN; long‐lasting in vivo activity (up to 1 week)	Immunogenicity concerns; requires de‐immunization strategies; clinical translation in early stages
Phage Therapy	Specific recognition and lysis of target bacteria; high selectivity preserves commensal microbiota	Selective targeting of IgAN‐associated *E. coli* strains	Highly selective; avoids antibiotic‐associated dysbiosis; favourable safety profile in other *E. coli* ‐related diseases	No direct clinical evidence in IgAN yet; requires identification of IgAN‐associated strains; phage cocktail optimization needed
Engineered Bacteria	Genetically modified probiotics (e.g., *E. coli* Nissle 1917) to sense inflammatory signals and deliver therapeutic molecules	Potential inhibition of pathogenic *E. coli* colonization; secretion of therapeutic proteins	In situ, continuous delivery of therapeutics; can be engineered for targeted release	Early research stage; long‐term colonization safety concerns; technical challenges in engineering and regulation

*Note:* ① Data and conclusions presented in this table are derived from clinical studies cited in this review (see corresponding references in the main text). ② Dietary intervention is typically used as an adjunctive therapy in combination with other regimens; its efficacy as a monotherapy has not yet been confirmed in randomized controlled trials. ③ Due to variations in intervention protocols, follow‐up durations, outcome measures, and patient populations across studies, direct comparisons of the efficacy of different intervention strategies should be interpreted with caution.④ Novel strategies (engineered IgA protease, phage therapy, engineered bacteria) are in preclinical/early clinical stages; direct evidence in IgAN is lacking.

Abbreviations: AK183, IgA protease derived from *Clostridium ramosum* ; *EcN*, *E. coli* Nissle 1917 (probiotic strain); FMT, faecal microbiota transplantation; GALT, gut‐associated lymphoid tissue; LBP‐EC01, phage therapy product for *E. coli* infections; SCFAs, short‐chain fatty acids.

### 5.1 Faecal Microbiota Transplantation

FMT has been investigated as a microbiota‐modulating intervention in IgAN, although its safety and efficacy remain under clinical evaluation (Monteiro et al. 2022). Clinical trials of FMT have been conducted in patients with refractory IgAN, and the results indicate that FMT is well tolerated in patients with IgAN, with no serious adverse events. Moreover, FMT has been shown to reduce proteinuria, and the decrease in *E. coli* abundance suggests that the underlying mechanism may involve modulation of the gut microbiota and metabolites, which in turn affects B‐cell immunity and ameliorates IgAN (Zhao et al. 2021, 2022; Zhi et al. 2024). However, current evidence has notable limitations, including small sample sizes and incomplete mechanistic investigations, warranting further large‐scale studies for validation. More broadly, recent studies of washed microbiota transplantation in other clinical settings have provided additional information regarding its feasibility and safety, although the applicability of these findings to IgAN remains to be established (Xu et al. 2026).

### 5.2 Probiotics and Dietary Intervention

Probiotics, favoured for their safety and ease of use, are a key strategy for intestinal intervention in IgAN. They inhibit *E. coli* overgrowth and ameliorate gut dysbiosis. Commonly studied probiotics include *Bifidobacterium* and *Clostridium butyricum* . Clinical studies show that supplementation with these probiotics reduces intestinal *E. coli* abundance, lowers serum endotoxin/LPS and pro‐inflammatory cytokines (e.g., TNF‐α, IL‐1β), and improves renal pathology and biochemical parameters (Tan et al. 2022; Dong, Yan, et al. 2025). Mechanistically, probiotics competitively inhibit pathogenic bacterial adhesion and proliferation, reducing virulence factors such as LPS (Mack et al. 1999; Han et al. 2021). Additionally, butyrate produced by *C. butyricum* repairs intestinal barrier function, limiting systemic LPS translocation (Liu et al. 2020; Ariyoshi et al. 2022; Yuan et al. 2025). However, strain‐specific effects, lack of standardized dosages, and insufficient long‐term safety data remain to be addressed.

In parallel, dietary interventions, particularly increased dietary fibre intake, may represent a feasible approach to modulating the gut microbiota and intestinal barrier function. A high‐fibre diet promotes the growth of butyrate‐producing bacteria (e.g., *Roseburia* and *Faecalibacterium*), enhances intestinal barrier function, and inhibits *E. coli* expansion (Liu et al. 2025). Furthermore, dietary fibre fermented by the gut microbiota produces short‐chain fatty acids (SCFAs, especially butyrate), which upregulate the expression of tight junction proteins and reduce inflammation (Pérez‐Reytor et al. 2021).

### 5.3 Engineered IgA Protease

In existing studies, the IgA protease AK183 derived from the commensal bacterium *Clostridium ramosum* was selected and fused with the Fc fragment of human IgG1. Using an *E. coli* expression system, the enzymatically active Fc‐AK183 fusion protein was successfully produced. Subsequent validation in mouse models showed that this protein exhibited in vivo activity lasting up to one week, effectively clearing circulating IgA as well as IgA and complement C3 already deposited in the glomeruli (Kosowska et al. 2002; Xie et al. 2022). This research finding provides a potential therapeutic approach for rapidly progressive IgAN.

### 5.4 Phage Therapy

Phage therapy specifically lyses target bacteria with minimal impact on commensal gut microbiota, offering high selectivity over broad‐spectrum antibiotics (Gordillo Altamirano and Barr 2019; Strathdee et al. 2023). It has shown safety and efficacy in urinary tract infections (e.g., LBP‐EC01, Phase I) (Kim et al. 2021) and intestinal infections (e.g., against *Shigella*) (Llanos‐Chea et al. 2019), but clinical studies in IgAN are lacking. Because *E. coli* is also a normal component of the gut microbiota, phage therapy in IgAN should not aim to eliminate all *E. coli* . Instead, its potential application would rely on the selective targeting of specific IgAN‐associated *E. coli* strains while preserving non‐target commensal bacteria, taking advantage of the strain‐level host specificity of bacteriophages (Gordillo Altamirano and Barr 2019; Gaborieau et al. 2024). In the future, developing specific phage cocktails targeting *E. coli* strains enriched in the gut of IgAN patients may provide a new direction for precision microbiota intervention in IgAN.

### 5.5 Engineered Bacteria

Advances in synthetic biology have enabled engineered bacteria to serve as programmable platforms for disease diagnosis and therapeutic delivery (Jin et al. 2025). The probiotic strain *E. coli* Nissle 1917 (EcN) has emerged as a promising chassis for therapeutic delivery (Lynch et al. 2023). Engineered EcN can sense inflammation‐associated intestinal signals and locally release therapeutic molecules, including anti‐TNF‐α nanobodies (Weibel et al. 2024). Inflammation‐targeting modifications can further improve the localized therapeutic activity of engineered EcN in the inflamed intestine (Hua et al. 2025).

Recent studies have also demonstrated the feasibility of engineering probiotic bacteria for selective antimicrobial delivery against specific disease‐associated microorganisms, supporting the broader potential of programmable microbiota‐targeted interventions (Choudhury et al. 2025). Although these approaches have not yet been specifically evaluated in IgAN, engineered probiotic strains could theoretically be designed to sense IgAN‐relevant intestinal signals and locally deliver therapeutic molecules to modulate intestinal inflammation, barrier function, or mucosal immune responses. Such a strategy may enable localized and controllable intervention while limiting systemic exposure. However, disease‐specific sensors and therapeutic payloads remain to be defined, and genetic stability, biocontainment, colonization, and long‐term safety require further investigation.

## 6 Challenges and Prospects

Despite growing evidence linking *E. coli* ‐associated dysbiosis to IgAN, several critical questions remain. First, it remains unclear whether all *E. coli* strains possess equivalent pathogenic potential or whether specific strains with distinct functional or metabolic characteristics are more closely associated with IgAN. Strain‐level resolution is needed to distinguish commensal strains from IgAN‐associated strains with potentially detrimental functional characteristics. Second, the interaction between *E. coli* and other gut microbiota members—particularly butyrate producers—warrants mechanistic studies to understand how ecological competition or cooperation influences disease progression. Third, the LPS‐induced NLRP3 inflammasome‐mediated pyroptosis pathway and S1P‐mediated remote regulation represent promising therapeutic targets, but their causal roles in human IgAN require validation in prospective cohorts and preclinical models. Fourth, for emerging precision strategies, key challenges include immunogenicity of bacterial‐derived enzymes (engineered IgA protease), identification of disease‐associated *E. coli* strains suitable for targeted phage development, and long‐term colonization safety of engineered bacteria.

## 7 Conclusion

*E. coli* represents one of the recurrent dysbiotic features of the gut microbiota in IgAN. Current evidence suggests that *E. coli* ‐associated dysbiosis may contribute to Gd‐IgA1 overproduction and renal injury through two major pathways. On one hand, LPS promotes B‐cell‐mediated overproduction of Gd‐IgA1, which deposits in the glomerular mesangium; simultaneously, LPS triggers NLRP3‐mediated pyroptosis in macrophages, accompanied by the release of IL‐1β/IL‐18, thereby exacerbating renal inflammation. On the other hand, *E. coli* ‐associated metabolic alterations, including increased circulating S1P, may promote mesangial proliferation and fibrosis, while indole‐ and TMA‐related pathways may contribute to tubulointerstitial injury. Together, these pathways provide a biologically plausible mechanistic framework linking gut dysbiosis to the gut–kidney axis in IgAN. Beyond conventional interventions, emerging precision therapeutic strategies—including engineered IgA protease, phage therapy, and engineered bacteria—offer new promise for mechanism‐informed intervention. Future research should focus on identifying key IgAN‐associated *E. coli* strains, elucidating their interaction mechanisms with the host and gut microbiota, and advancing these precision strategies toward clinical translation.

## Author Contributions

**Yuxin Liu:** writing – original draft, data curation. **Xiaoxuan Ning:** data curation, writing – original draft. **Yichen Liu:** data curation, writing – review and editing. **Zixian Yu:** writing – review and editing, data curation. **Jie Liu:** validation. **Xiaoqin Xu:** visualization. **Jin Zhao:** writing – review and editing, supervision. **Shiren Sun:** conceptualization, project administration, writing – review and editing, supervision.

## Funding

This work was supported by the National Natural Science Foundation of China (82470735), the Shaanxi Provincial Health Commission (2023TD‐02), the Key Research and Development Program of Shaanxi Province (2023‐ZDLSF‐15) and the Key Research and Development Projects of Shaanxi Province (2022ZDLSF03‐12).

## Disclosure

The authors have nothing to report.

## Ethics Statement

The authors have nothing to report.

## Consent

The authors have nothing to report.

## Conflicts of Interest

The authors declare no conflicts of interest.

## Supporting information


**Data S1:** Supporting Information.

## Data Availability

Data sharing not applicable to this article as no datasets were generated or analysed during the current study.

## References

[mbt270455-bib-0001] Ariyoshi, T. , M. Hagihara , M. Takahashi , and H. Mikamo . 2022. “Effect of *Clostridium butyricum* on Gastrointestinal Infections.” Biomedicine 10: 483. 10.3390/biomedicines10020483 PMC8962260 35203691

[mbt270455-bib-0002] Bachmann, P. , P. Afanasyev , D. Boehringer , and R. Glockshuber . 2025. “Structures of the *Escherichia coli* Type 1 Pilus During Pilus Rod Assembly and After Assembly Termination.” Nature Communications 16: 4988. 10.1038/s41467-025-60325-z PMC12122975 40442073

[mbt270455-bib-0003] Bäumler, A. , M. Byndloss , and Y. Litvak . 2018. “Colonocyte Metabolism Shapes the Gut Microbiota.” Science 362: eaat9076. 10.1126/science.aat9076 PMC6296223 30498100

[mbt270455-bib-0004] Belkaid, Y. , and T. W. Hand . 2014. “Role of the Microbiota in Immunity and Inflammation.” Cell 157: 121–141. 10.1016/j.cell.2014.03.011 PMC4056765 24679531

[mbt270455-bib-0005] Berger, J. , and N. Hinglais . 1968. “Intercapillary Deposits of IgA‐IgG.” Journal D'urologie et de Néphrologie 74: 694–695. 4180586

[mbt270455-bib-0006] Chen, X. , M. Li , S. Zhu , et al. 2023. “Proteomic Profiling of IgA Nephropathy Reveals Distinct Molecular Prognostic Subtypes.” iScience 26: 105961. 10.1016/j.isci.2023.105961 PMC9984961 36879796

[mbt270455-bib-0007] Cheng, Y. , X. Xiao , J. Fu , X. Zong , Z. Lu , and Y. Wang . 2024. “ *Escherichia coli* K88 Activates NLRP3 Inflammasome‐Mediated Pyroptosis In Vitro and In Vivo.” Biochemistry and Biophysics Reports 38: 101665. 10.1016/j.bbrep.2024.101665 PMC10900769 38419757

[mbt270455-bib-0008] Choudhury, A. , C. Scano , A. Barton , C. M. Kearney , and K. L. Greathouse . 2025. “Precision Antimicrobial Therapy Against *Fusobacterium nucleatum* Using Bioengineered Probiotics Expressing Guided Antimicrobial Peptides (gAMPs).” Microbial Biotechnology 18: e70241. 10.1111/1751-7915.70241 PMC12690405 41368940

[mbt270455-bib-0009] Clements, A. , J. C. Young , N. Constantinou , and G. Frankel . 2012. “Infection Strategies of Enteric Pathogenic *Escherichia coli* .” Gut Microbes 3: 71–87. 10.4161/gmic.19182 PMC3370951 22555463

[mbt270455-bib-0010] Collins, S. M. , E. Denou , E. F. Verdu , and P. Bercik . 2009. “The Putative Role of the Intestinal Microbiota in the Irritable Bowel Syndrome.” Digestive and Liver Disease 41: 850–853. 10.1016/j.dld.2009.07.023 19740713

[mbt270455-bib-0011] Conway, T. , and P. S. Cohen . 2015. “Commensal and Pathogenic *Escherichia coli* Metabolism in the Gut.” Microbiology Spectrum 3: 3.3.24. 10.1128/microbiolspec.MBP-0006-2014 PMC4510460 26185077

[mbt270455-bib-0012] Coppo, R. 2018. “The Gut‐Renal Connection in IgA Nephropathy.” Seminars in Nephrology 38: 504–512. 10.1016/j.semnephrol.2018.05.020 30177022

[mbt270455-bib-0013] Deng, Y. , Z. Nong , M. Wei , et al. 2024. “Characteristics and Function of the Gut Microbiota in Patients With IgA Nephropathy via Metagenomic Sequencing Technology.” Renal Failure 46: 2393754. 10.1080/0886022X.2024.2393754 PMC11346320 39177227

[mbt270455-bib-0014] Di Giorgio, C. , R. Roselli , M. Biagioli , et al. 2023. “Modeling Inflammatory Bowel Disease by Intestinal Organoids.” Recent Advances in Inflammation & Allergy Drug Discovery 17: 39–53. 10.2174/2772270817666221121143853 36411558

[mbt270455-bib-0015] Dong, R. , M. Bai , J. Zhao , D. Wang , X. Ning , and S. Sun . 2020. “A Comparative Study of the Gut Microbiota Associated With Immunoglobulin a Nephropathy and Membranous Nephropathy.” Frontiers in Cellular and Infection Microbiology 10: 557368. 10.3389/fcimb.2020.557368 PMC7606180 33194798

[mbt270455-bib-0016] Dong, Y. , G. Yan , Y. Zhang , Y. Zhou , and J. Shang . 2025. “Gut Microbiota as a Predictive Tool for Outcomes in IgA Nephropathy.” Renal Failure 47: 2514184. 10.1080/0886022X.2025.2514184 PMC12243011 40629894

[mbt270455-bib-0017] Dong, Z. , R. Zhang , L. Shen , et al. 2025. “Gut Microbiota and Immunoglobulin A Nephropathy: Exploration of Dietary Intervention and Treatment Strategies.” Food Science & Nutrition 13: e70218. 10.1002/fsn3.70218 PMC12045934 40321610

[mbt270455-bib-0018] Drexler, Y. , J. Molina , A. Mitrofanova , A. Fornoni , and S. Merscher . 2021. “Sphingosine‐1‐Phosphate Metabolism and Signaling in Kidney Diseases.” Journal of the American Society of Nephrology 32: 9–31. 10.1681/ASN.2020050697 PMC7894665 33376112

[mbt270455-bib-0019] Ebbestad, R. , M. Sanaei Nurmi , and S. Lundberg . 2022. “Long‐Term Outcomes of Patients With IgA Nephropathy Categorized by the International IgAN Risk Prediction Tool and by the Degree of Hematuria at Diagnosis.” Nephron 146: 573–583. 10.1159/000525001 PMC9808712 35671707

[mbt270455-bib-0020] Elliott, S. J. , E. O. Krejany , J. L. Mellies , R. M. Robins‐Browne , C. Sasakawa , and J. B. Kaper . 2001. “EspG, a Novel Type III System‐Secreted Protein From Enteropathogenic *Escherichia coli* With Similarities to VirA of *Shigella flexneri* .” Infection and Immunity 69: 4027–4033. 10.1128/IAI.69.6.4027-4033.2001 PMC98465 11349072

[mbt270455-bib-0021] Fu, Y. , H. Kim , D. S. Lee , et al. 2025. “Structural Insight Into TLR4/MD‐2 Activation by Synthetic LPS Mimetics With Distinct Binding Modes.” Nature Communications 16: 4164. 10.1038/s41467-025-59550-3 PMC12053604 40325026

[mbt270455-bib-0022] Gaborieau, B. , H. Vaysset , F. Tesson , et al. 2024. “Prediction of Strain Level Phage‐Host Interactions Across the Escherichia Genus Using Only Genomic Information.” Nature Microbiology 9: 2847–2861. 10.1038/s41564-024-01832-5 39482383

[mbt270455-bib-0023] Gao, L. , H. Li , X. Liu , et al. 2024. “Humoral Immune Responses Primed by the Alteration of Gut Microbiota Were Associated With Galactose‐Deficient IgA1 Production in IgA Nephropathy.” Frontiers in Immunology 15: 1415026. 10.3389/fimmu.2024.1415026 PMC11298704 39104521

[mbt270455-bib-0024] Gerasimidis, K. , K. Bryden , X. Chen , et al. 2019. “The Impact of Food Additives, Artificial Sweeteners and Domestic Hygiene Products on the Human Gut Microbiome and Its Fibre Fermentation Capacity.” European Journal of Nutrition 59: 3213–3230. 10.1007/s00394-019-02161-8 PMC7501109 31853641

[mbt270455-bib-0025] Gordillo Altamirano, F. L. , and J. J. Barr . 2019. “Phage Therapy in the Postantibiotic Era.” Clinical Microbiology Reviews 32: e00066‐18. 10.1128/CMR.00066-18 PMC6431132 30651225

[mbt270455-bib-0026] Hamed, S. A. , A. Mohan , S. Navaneetha Krishnan , et al. 2023. “Butyrate Reduces Adherent‐Invasive *E. coli* ‐Evoked Disruption of Epithelial Mitochondrial Morphology and Barrier Function: Involvement of Free Fatty Acid Receptor 3.” Gut Microbes 15: 2281011. 10.1080/19490976.2023.2281011 PMC10730202 38078655

[mbt270455-bib-0027] Han, S. , Y. Lu , J. Xie , et al. 2021. “Probiotic Gastrointestinal Transit and Colonization After Oral Administration: A Long Journey.” Frontiers in Cellular and Infection Microbiology 11: 609722. 10.3389/fcimb.2021.609722 PMC8006270 33791234

[mbt270455-bib-0028] Hayashi, F. , K. D. Smith , A. Ozinsky , et al. 2001. “The Innate Immune Response to Bacterial Flagellin Is Mediated by Toll‐Like Receptor 5.” Nature 410: 1099–1103. 10.1038/35074106 11323673

[mbt270455-bib-0029] Hornef, M. W. , B. H. Normark , A. Vandewalle , and S. Normark . 2003. “Intracellular Recognition of Lipopolysaccharide by Toll‐Like Receptor 4 in Intestinal Epithelial Cells.” Journal of Experimental Medicine 198: 1225–1235. 10.1084/jem.20022194 PMC2194240 14568981

[mbt270455-bib-0030] Hu, X. , J. Du , Y. Xie , et al. 2020. “Fecal Microbiota Characteristics of Chinese Patients With Primary IgA Nephropathy: A Cross‐Sectional Study.” BMC Nephrology 21: 97. 10.1186/s12882-020-01741-9 PMC7071635 32169051

[mbt270455-bib-0031] Hua, S. , K. Li , P. Shang , et al. 2025. “An Inflammation‐Targeting Engineered Probiotic *Escherichia coli* Nissle 1917 With High Anti‐TNF‐α Nanobody Secretion Efficacy Alleviates Ulcerative Colitis.” Advanced Science 12: e12360. 10.1002/advs.202512360 PMC12697777 41017573

[mbt270455-bib-0032] Jin, K. , Y. Huang , H. Che , and Y. Wu . 2025. “Engineered Bacteria for Disease Diagnosis and Treatment Using Synthetic Biology.” Microbial Biotechnology 18: e70080. 10.1111/1751-7915.70080 PMC11725985 39801378

[mbt270455-bib-0033] Kalkan, A. E. , M. N. BinMowyna , A. Raposo , et al. 2025. “Beyond the Gut: Unveiling Butyrate's Global Health Impact Through Gut Health and Dysbiosis‐Related Conditions: A Narrative Review.” Nutrients 17: 1305. 10.3390/nu17081305 PMC12029953 40284169

[mbt270455-bib-0034] Kalniņš, G. , J. Kuka , S. Grinberga , et al. 2015. “Structure and Function of CutC Choline Lyase From Human Microbiota Bacterium *Klebsiella pneumoniae* .” Journal of Biological Chemistry 290: 21732–21740. 10.1074/jbc.M115.670471 PMC4571895 26187464

[mbt270455-bib-0035] Kim, P. , A. Sanchez , J. Kime , and D. Ousterout . 2021. “1083. Phase 1b Results of Pharmacokinetics, Pharmacodynamics, and Safety for LBP‐EC01, a CRISPR‐Cas3 Enhanced Bacteriophage Cocktail Targeting *Escherichia coli* That Cause Urinary Tract Infections.” Open Forum Infectious Diseases 8: S633. 10.1016/S1473-3099(24)00424-9 39134085

[mbt270455-bib-0036] Kiryluk, K. , J. Novak , and A. G. Gharavi . 2013. “Pathogenesis of Immunoglobulin A Nephropathy: Recent Insight From Genetic Studies.” Annual Review of Medicine 64: 339–356. 10.1146/annurev-med-041811-142014 PMC3921622 23072577

[mbt270455-bib-0037] Koch, L. , D. Frommhold , K. Buschmann , N. Kuss , J. Poeschl , and P. Ruef . 2014. “LPS‐ and LTA‐Induced Expression of IL‐6 and TNF‐α in Neonatal and Adult Blood: Role of MAPKs and NF‐κB.” Mediators of Inflammation 2014: 283126. 10.1155/2014/283126 PMC4229971 25530682

[mbt270455-bib-0038] Korotkova, N. , E. Cota , Y. Lebedin , et al. 2006. “A Subfamily of Dr Adhesins of *Escherichia coli* Bind Independently to Decay‐Accelerating Factor and the N‐Domain of Carcinoembryonic Antigen.” Journal of Biological Chemistry 281: 29120–29130. 10.1074/jbc.M605681200 PMC2629542 16882658

[mbt270455-bib-0039] Kosowska, K. , J. Reinholdt , L. K. Rasmussen , et al. 2002. “The *Clostridium ramosum* IgA Proteinase Represents a Novel Type of Metalloendopeptidase.” Journal of Biological Chemistry 277: 11987–11994. 10.1074/jbc.M110883200 11815614

[mbt270455-bib-0040] Krogfelt, K. A. , H. Bergmans , and P. Klemm . 1990. “Direct Evidence That the FimH Protein Is the Mannose‐Specific Adhesin of *Escherichia coli* Type 1 Fimbriae.” Infection and Immunity 58: 1995–1998. 10.1128/iai.58.6.1995-1998.1990 PMC258756 1971261

[mbt270455-bib-0041] Książek, M. , M. Chacińska , A. Chabowski , and M. Baranowski . 2015. “Sources, Metabolism, and Regulation of Circulating Sphingosine‐1‐Phosphate.” Journal of Lipid Research 56: 1271–1281. 10.1194/jlr.R059543 PMC4479332 26014962

[mbt270455-bib-0066] LaBouyer, M. , G. Holtrop , G. Horgan , et al. 2022. “Higher Total Faecal Short Chain Fatty Concentrations Correlate With Increasing Proportions of Butyrate and Decreasing Proportions of Branched Chain fatty acids across multiple human studies.” Gut Microbiome 3: e2. 10.1017/gmb.2022.1 PMC11406374 39295782

[mbt270455-bib-0042] Lee, J. Y. , S. P. Mahan , T. Parente de Carvalho , et al. 2026. “Host‐Derived Nitrate Fuels Indole Production by *Escherichia coli* to Drive Chronic Kidney Disease Progression.” Science 391: 1250–1255. 10.1126/science.ady5217 PMC13004173 41855331

[mbt270455-bib-0043] Lee, M. , H. Suzuki , Y. Nihei , K. Matsuzaki , and Y. Suzuki . 2023. “Ethnicity and IgA Nephropathy: Worldwide Differences in Epidemiology, Timing of Diagnosis, Clinical Manifestations, Management and Prognosis.” Clinical Kidney Journal 16: ii1–ii8. 10.1093/ckj/sfad199 PMC10695519 38053973

[mbt270455-bib-0044] Lim, B. J. , D. Lee , S. W. Hong , and H. J. Jeong . 2011. “Toll‐Like Receptor 4 Signaling Is Involved in IgA‐Stimulated Mesangial Cell Activation.” Yonsei Medical Journal 52: 610–615. 10.3349/ymj.2011.52.4.610 PMC3104456 21623603

[mbt270455-bib-0045] Liu, C. , J. Wang , L. Lei , L. Li , and X. Yuan . 2025. “Gut Microbiota Therapy for Chronic Kidney Disease.” Frontiers in Immunology 16: 1660226. 10.3389/fimmu.2025.1660226 PMC12457294 41000396

[mbt270455-bib-0046] Liu, M. , Y. Lu , G. Xue , et al. 2024. “Role of Short‐Chain Fatty Acids in Host Physiology.” Animal Models and Experimental Medicine 7: 641–652. 10.1002/ame2.12464 PMC11528394 38940192

[mbt270455-bib-0047] Liu, M. , W. Xie , X. Wan , and T. Deng . 2020. “ *Clostridium butyricum* Protects Intestinal Barrier Function via Upregulation of Tight Junction Proteins and Activation of the Akt/mTOR Signaling Pathway in a Mouse Model of Dextran Sodium Sulfate‐Induced Colitis.” Experimental and Therapeutic Medicine 20: 10. 10.3892/etm.2020.9138 PMC7471846 32934675

[mbt270455-bib-0048] Llanos‐Chea, A. , R. J. Citorik , K. P. Nickerson , et al. 2019. “Bacteriophage Therapy Testing Against Shigella flexneri in a Novel Human Intestinal Organoid‐Derived Infection Model.” Journal of Pediatric Gastroenterology and Nutrition 68: 509–516. 10.1097/MPG.0000000000002203 PMC6939622 30418409

[mbt270455-bib-0049] Lopes, J. , I. Pereira , J. Gameiro , and M. Pereira . 2025. “The Gut Microbiome as a Potential Therapeutic Target in IgA Nephropathy.” Nephrology, Dialysis, Transplantation 40: 641–650. 10.1093/ndt/gfae274 39663206

[mbt270455-bib-0050] Louis, P. , and H. J. Flint . 2017. “Formation of Propionate and Butyrate by the Human Colonic Microbiota.” Environmental Microbiology 19, no. 1: 29–41. 10.1111/1462-2920.13589 27928878

[mbt270455-bib-0051] Lynch, J. P. , C. González‐Prieto , A. Z. Reeves , et al. 2023. “Engineered *Escherichia coli* for the In Situ Secretion of Therapeutic Nanobodies in the Gut.” Cell Host & Microbe 31: 634–649.e638. 10.1016/j.chom.2023.03.007 PMC10101937 37003258

[mbt270455-bib-0052] Maceyka, M. , and S. Spiegel . 2014. “Sphingolipid Metabolites in Inflammatory Disease.” Nature 510: 58–67. 10.1038/nature13475 PMC4320971 24899305

[mbt270455-bib-0053] Mack, D. R. , S. Michail , S. Wei , L. McDougall , and M. A. Hollingsworth . 1999. “Probiotics Inhibit Enteropathogenic *E. coli* Adherence In Vitro by Inducing Intestinal Mucin Gene Expression.” American Journal of Physiology 276: G941–G950. 10.1152/ajpgi.1999.276.4.G941 10198338

[mbt270455-bib-0054] Marchesi, J. R. , D. H. Adams , F. Fava , et al. 2016. “The Gut Microbiota and Host Health: A New Clinical Frontier.” Gut 65: 330–339. 10.1136/gutjnl-2015-309990 PMC4752653 26338727

[mbt270455-bib-0055] Miller, E. , T. Garcia , S. Hultgren , and A. F. Oberhauser . 2006. “The Mechanical Properties of *E. coli* Type 1 Pili Measured by Atomic Force Microscopy Techniques.” Biophysical Journal 91: 3848–3856. 10.1529/biophysj.106.088989 PMC1630485 16950852

[mbt270455-bib-0056] Monteiro, R. C. , D. Rafeh , and P. J. Gleeson . 2022. “Is There a Role for Gut Microbiome Dysbiosis in IgA Nephropathy?” Microorganisms 10: 683. 10.3390/microorganisms10040683 PMC9031807 35456735

[mbt270455-bib-0057] Pérez‐Reytor, D. , C. Puebla , E. Karahanian , and K. García . 2021. “Use of Short‐Chain Fatty Acids for the Recovery of the Intestinal Epithelial Barrier Affected by Bacterial Toxins.” Frontiers in Physiology 12: 650313. 10.3389/fphys.2021.650313 PMC8181404 34108884

[mbt270455-bib-0058] Popova, A. , K. Rācenis , M. Brīvība , et al. 2025. “Reduced Butyrate‐Producing Bacteria and Altered Metabolic Pathways in the Gut Microbiome of Immunoglobulin A Nephropathy Patients.” Scientific Reports 15: 28011. 10.1038/s41598-025-13629-5 PMC12313972 40745465

[mbt270455-bib-0059] Remuzzi, G. , A. Benigni , and A. Remuzzi . 2006. “Mechanisms of Progression and Regression of Renal Lesions of Chronic Nephropathies and Diabetes.” Journal of Clinical Investigation 116: 288–296. 10.1172/JCI27699 PMC1359063 16453013

[mbt270455-bib-0060] Roberts, L. E. , C. E. C. Williams , L. Oni , J. Barratt , and H. Selvaskandan . 2024. “IgA Nephropathy: Emerging Mechanisms of Disease.” Indian Journal of Nephrology 34: 297–309. 10.25259/ijn_425_23 PMC11326799 39156850

[mbt270455-bib-0061] Rovin, B. H. , S. G. Adler , J. Barratt , et al. 2021. “KDIGO 2021 Clinical Practice Guideline for the Management of Glomerular Diseases.” Kidney International 100: S1–S276. 10.1016/j.kint.2021.05.021 34556256

[mbt270455-bib-0062] Saha, S. , E. Pupo , A. Zariri , and P. van der Ley . 2022. “Lipid A Heterogeneity and Its Role in the Host Interactions With Pathogenic and Commensal Bacteria.” Microlife 3: uqac011. 10.1093/femsml/uqac011 PMC10117875 37223360

[mbt270455-bib-0063] Sanz, Y. 2010. “Effects of a Gluten‐Free Diet on Gut Microbiota and Immune Function in Healthy Adult Humans.” Gut Microbes 1: 135–137. 10.4161/gmic.1.3.11868 PMC3023594 21327021

[mbt270455-bib-0064] Schwalm, S. , R. Manaila , A. Oftring , L. Schaefer , S. von Gunten , and J. Pfeilschifter . 2024. “The Contribution of the Sphingosine 1‐Phosphate Signaling Pathway to Chronic Kidney Diseases: Recent Findings and New Perspectives.” Pflügers Archiv 476: 1845–1861. 10.1007/s00424-024-03029-5 PMC11582123 39384640

[mbt270455-bib-0065] Schwalm, S. , J. Pfeilschifter , and A. Huwiler . 2013. “Sphingosine‐1‐Phosphate: A Janus‐Faced Mediator of Fibrotic Diseases.” Biochimica et Biophysica Acta 1831: 239–250. 10.1016/j.bbalip.2012.07.022 22889995

[mbt270455-bib-0067] Sekirov, I. , S. L. Russell , L. C. M. Antunes , and B. B. Finlay . 2010. “Gut Microbiota in Health and Disease.” Physiological Reviews 90, no. 3: 859–904. 10.1152/physrev.00045.2009 20664075

[mbt270455-bib-0068] Shen, Y. , N. Fan , S. X. Ma , X. Cheng , X. Yang , and G. Wang . 2025. “Gut Microbiota Dysbiosis: Pathogenesis, Diseases, Prevention, and Therapy.” MedComm 6: e70168. 10.1002/mco2.70168 PMC12006732 40255918

[mbt270455-bib-0069] Singh, A. P. , S. Sharma , K. Pagarware , et al. 2018. “Enteropathogenic *E. coli* Effectors EspF and Map Independently Disrupt Tight Junctions Through Distinct Mechanisms Involving Transcriptional and Post‐Transcriptional Regulation.” Scientific Reports 8: 3719. 10.1038/s41598-018-22017-1 PMC5829253 29487356

[mbt270455-bib-0070] Sterling, K. G. , G. K. Dodd , S. Alhamdi , et al. 2022. “Mucosal Immunity and the Gut‐Microbiota‐Brain‐Axis in Neuroimmune Disease.” International Journal of Molecular Sciences 23: 13328. 10.3390/ijms232113328 PMC9655506 36362150

[mbt270455-bib-0071] Stoneman, S. , J. W. Teh , and M. M. O'Shaughnessy . 2026. “IgA Nephropathy in Adults.” JAMA 335, no. 9: 799–813. 10.1001/jama.2025.25020 41587026

[mbt270455-bib-0072] Strathdee, S. A. , G. F. Hatfull , V. K. Mutalik , and R. T. Schooley . 2023. “Phage Therapy: From Biological Mechanisms to Future Directions.” Cell 186, no. 1: 17–31. 10.1016/j.cell.2022.11.017 PMC9827498 36608652

[mbt270455-bib-0073] Tan, J. , L. Dong , Z. Jiang , et al. 2022. “Probiotics Ameliorate IgA Nephropathy by Improving Gut Dysbiosis and Blunting NLRP3 Signaling.” Journal of Translational Medicine 20: 382. 10.1186/s12967-022-03585-3 PMC9422169 36038927

[mbt270455-bib-0074] Tang, W. H. , and S. L. Hazen . 2014. “The Contributory Role of Gut Microbiota in Cardiovascular Disease.” Journal of Clinical Investigation 124: 4204–4211. 10.1172/JCI72331 PMC4215189 25271725

[mbt270455-bib-0075] Tang, Y. , Y. Li , X. Yang , et al. 2026. “Intestinal Metabolite TMAO Promotes CKD Progression by Stimulating Macrophage M2 Polarization Through Histone H4 Lysine 12 Lactylation.” Cell Death and Differentiation 33: 314–326. 10.1038/s41418-025-01554-z PMC12881611 40830246

[mbt270455-bib-0076] Tang, Y. , Y. Xiao , H. He , et al. 2023. “Aberrant Gut Microbiome Contributes to Barrier Dysfunction, Inflammation, and Local Immune Responses in IgA Nephropathy.” Kidney & Blood Pressure Research 48: 261–276. 10.1159/000528973 PMC10158088 36878203

[mbt270455-bib-0077] Tang, Y. , D. Zhang , L. Huang , et al. 2025. “Gut Microbial Metabolite, Sphingosine‐1‐Phosphate (S1P), Drives Mesangial Cell Phenotypic Transformation and Accelerates Progression of IgA Nephropathy via CCL2‐MET‐FAK Pathway.” Current Research in Microbial Sciences 9: 100494. 10.1016/j.crmicr.2025.100494 PMC12590225 41209721

[mbt270455-bib-0078] Tominaga, A. , M. A. Mahmoud , A. A. Al Mamun , and T. Mukaihara . 2001. “Characterization of Cryptic Flagellin Genes in Shigella Boydii and *Shigella dysenteriae* .” Genes & Genetic Systems 76: 111–120. 10.1266/ggs.76.111 11434456

[mbt270455-bib-0079] Wang, J. , M. Liang , J. Xu , et al. 2014. “Renal Expression of Advanced Oxidative Protein Products Predicts Progression of Renal Fibrosis in Patients With IgA Nephropathy.” Laboratory Investigation 94: 966–977. 10.1038/labinvest.2014.90 25068662

[mbt270455-bib-0080] Wang, X. , X. J. Zhou , X. Qiao , M. Falchi , J. Liu , and H. Zhang . 2025. “The Evolving Understanding of Systemic Mechanisms in Organ‐Specific IgA Nephropathy: A Focus on Gut‐Kidney Crosstalk.” Theranostics 15: 656–681. 10.7150/thno.104631 PMC11671385 39744688

[mbt270455-bib-0081] Weibel, N. , M. Curcio , A. Schreiber , et al. 2024. “Engineering a Novel Probiotic Toolkit in *Escherichia coli* Nissle 1917 for Sensing and Mitigating Gut Inflammatory Diseases.” ACS Synthetic Biology 13: 2376–2390. 10.1021/acssynbio.4c00036 PMC11334186 39115381

[mbt270455-bib-0082] Wu, G. , T. Xu , N. Zhao , et al. 2024. “A Core Microbiome Signature as an Indicator of Health.” Cell 187: 6550–6565.e6511. 10.1016/j.cell.2024.09.019 39378879

[mbt270455-bib-0083] Wu, J. , T. Xie , Z. Zhang , et al. 2026. “Absence of Glomerular IgA1 Deposition Despite Overexpression of Galactose‐Deficient IgA1 in the B Cell c1galt1 Knockout Mouse.” Kidney International 110: 681–697. 10.1016/j.kint.2026.02.034 41905596

[mbt270455-bib-0084] Wu, X. , L. Zhao , K. Li , and J. Yang . 2022. “The Role of NLRP3 Inflammasome in IgA Nephropathy.” Medicina (Kaunas, Lithuania) 59: 82. 10.3390/medicina59010082 PMC9866943 36676706

[mbt270455-bib-0085] Xie, X. , J. Li , P. Liu , et al. 2022. “Chimeric Fusion Between *Clostridium ramosum* IgA Protease and IgG Fc Provides Long‐Lasting Clearance of IgA Deposits in Mouse Models of IgA Nephropathy.” Journal of the American Society of Nephrology 33: 918–935. 10.1681/ASN.2021030372 PMC9063903 35172987

[mbt270455-bib-0086] Xu, J. , A. Diao , R. Ai , et al. 2026. “Feasibility and Safety of Washed Microbiota Transplantation as Salvage Therapy for Refractory Non‐CDI Gut Pathogens in Critically Ill Patients.” Microbial Biotechnology 19: e70431. 10.1111/1751-7915.70431 PMC13473751 42596514

[mbt270455-bib-0087] Xu, L. , C. Wu , and G. Li . 2025. “Prevalence and Trend of Biopsy‐Proven IgA Nephropathy in China: A Systematic Review.” Journal of Nephrology 38: 1557–1564. 10.1007/s40620-025-02261-1 40138167

[mbt270455-bib-0088] Yuan, M. , K. Gao , K. Peng , S. Bi , X. Cui , and Y. Liu . 2025. “A Review of Nutritional Regulation of Intestinal Butyrate Synthesis: Interactions Between Dietary Polysaccharides and Proteins.” Food 14: 3649. 10.3390/foods14213649 PMC12607390 41227622

[mbt270455-bib-0089] Zhan, Z. , H. Tang , Y. Zhang , X. Huang , and M. Xu . 2022. “Potential of Gut‐Derived Short‐Chain Fatty Acids to Control Enteric Pathogens.” Frontiers in Microbiology 13: 976406. 10.3389/fmicb.2022.976406 PMC9530198 36204607

[mbt270455-bib-0090] Zhang, R. , X. Ji , Y. Tang , et al. 2025. “Chitooligosaccharides Ameliorate Glomerular Mesangial Proliferation and Inflammation in IgA Nephropathy by Blocking S1PR1/S1PR3 Pathway.” Food Science and Human Wellness (doi: 1026599/FSHW.2025.9250563).

[mbt270455-bib-0091] Zhang, R. , Y. Tang , X. Feng , et al. 2025. “Targeted Modulation of Intestinal Barrier and Mucosal Immune‐Related Microbiota Attenuates IgA Nephropathy Progression.” Gut Microbes 17: 2458184. 10.1080/19490976.2025.2458184 PMC11776482 39875350

[mbt270455-bib-0092] Zhang, W. , E.‐K. An , S.‐J. Kim , H.‐B. Park , P. C. W. Lee , and J.‐O. Jin . 2023. “ *Escherichia coli* Adhesion Protein FimH Exacerbates Colitis via CD11b+CD103‐ Dendritic Cell Activation.” Frontiers in Immunology 14: 1284770. 10.3389/fimmu.2023.1284770 PMC10703180 38077339

[mbt270455-bib-0093] Zhang, X. , J. K. Ritter , and N. Li . 2018. “Sphingosine‐1‐Phosphate Pathway in Renal Fibrosis.” American Journal of Physiology. Renal Physiology 315: F752–f756. 10.1152/ajprenal.00596.2017 PMC6230725 29631354

[mbt270455-bib-0094] Zhang, Y. , X. Han , T. Feng , et al. 2025. “Gut‐Microbiota‐Derived Indole Sulfate Promotes Heart Failure in Chronic Kidney Disease.” Cell Host & Microbe 33: 1715–1730.e1715. 10.1016/j.chom.2025.08.014 40961933

[mbt270455-bib-0095] Zhang, Y.‐M. , X.‐J. Zhou , and H. Zhang . 2017. “What Genetics Tells us About the Pathogenesis of IgA Nephropathy: The Role of Immune Factors and Infection.” Kidney International Reports 2: 318–331. 10.1016/j.ekir.2017.02.005 PMC5678660 29142962

[mbt270455-bib-0098] Zhang, Z. , L. Taylor., N. Shommu , et al. 2020. “A Diversified Dietary Pattern Is Associated With a Balanced Gut Microbial Composition of Faecalibacterium and Escherichia/Shigella in Patients With Crohn's Disease in Remission.” Journal of Crohn's & Colitis 14, no. 11: 1547–1557. 10.1093/ecco-jcc/jjaa084 32343765

[mbt270455-bib-0096] Zhao, J. , M. Bai , X. Ning , et al. 2022. “Expansion of Escherichia‐Shigella in Gut Is Associated With the Onset and Response to Immunosuppressive Therapy of IgA Nephropathy.” Journal of the American Society of Nephrology 33: 2276–2292. 10.1681/ASN.2022020189 PMC9731625 36041791

[mbt270455-bib-0097] Zhao, J. , M. Bai , X. Yang , Y. Wang , R. Li , and S. Sun . 2021. “Alleviation of Refractory IgA Nephropathy by Intensive Fecal Microbiota Transplantation: The First Case Reports.” Renal Failure 43: 928–933. 10.1080/0886022X.2021.1936038 PMC8901287 34134605

[mbt270455-bib-0099] Zhi, W. , A. Li , Q. Wang , et al. 2024. “Safety and Efficacy Assessment of Fecal Microbiota Transplantation as an Adjunctive Treatment for IgA Nephropathy: An Exploratory Clinical Trial.” Scientific Reports 14: 22935. 10.1038/s41598-024-74171-4 PMC11446926 39358432

[mbt270455-bib-0100] Zhong, Z. , J. Tan , L. Tan , et al. 2020. “Modifications of Gut Microbiota Are Associated With the Severity of IgA Nephropathy in the Chinese Population.” International Immunopharmacology 89: 107085. 10.1016/j.intimp.2020.107085 33068859

[mbt270455-bib-0101] Zhou, N. , Y. Shen , L. Fan , et al. 2020. “The Characteristics of Intestinal‐Barrier Damage in Rats With IgA Nephropathy.” American Journal of the Medical Sciences 359: 168–176. 10.1016/j.amjms.2019.11.011 32089158

[mbt270455-bib-0102] Zhou, Z. , H. Jin , H. Ju , M. Sun , H. Chen , and L. Li . 2022. “Circulating Trimethylamine‐N‐Oxide and Risk of All‐Cause and Cardiovascular Mortality in Patients With Chronic Kidney Disease: A Systematic Review and Meta‐Analysis.” Frontiers in Medicine 9: 828343. 10.3389/fmed.2022.828343 PMC9012260 35433743

[mbt270455-bib-0103] Zhu, W. , J. A. Buffa , Z. Wang , et al. 2018. “Flavin Monooxygenase 3, the Host Hepatic Enzyme in the Metaorganismal Trimethylamine N‐Oxide‐Generating Pathway, Modulates Platelet Responsiveness and Thrombosis Risk.” Journal of Thrombosis and Haemostasis 16: 1857–1872. 10.1111/jth.14234 PMC6156942 29981269

[mbt270455-bib-0104] Zhu, Y. , H. He , W. Sun , et al. 2024. “IgA Nephropathy: Gut Microbiome Regulates the Production of Hypoglycosilated IgA1 via the TLR4 Signaling Pathway.” Nephrology, Dialysis, Transplantation 39: 1624–1641. 10.1093/ndt/gfae052 PMC11427068 38402460

