# Supplementary material for: Potential Roles of Escherichia coli in IgA Nephropathy: Evidence, Mechanistic Hypotheses and Microbiota‐Targeted Strategies
Source: Microb Biotechnol. 2026 Oct 12;19(10):e70455. doi: 10.1111/1751-7915.70455 (PMC13655974; doi:10.1111/1751-7915.70455)
Supplement: Supplementary file 1 [file MBT2-19-e70455-s001.docx]

**Literature Search Strategy**

This narrative review was informed by a structured literature search of PubMed/MEDLINE and Web of Science, conducted up to July 31, 2026. The search combined terms related to IgA nephropathy (“IgA nephropathy” OR “IgAN”) with terms related to *Escherichia coli* and the gut microbiota (“Escherichia coli” OR “E. coli” OR “Escherichia-Shigella” OR “gut microbiota” OR “gut microbiome”), together with mechanism- and intervention-related terms including “lipopolysaccharide,” “LPS,” “virulence,” “intestinal barrier,” “probiotics,” “fecal microbiota transplantation,” “phage therapy,” and “engineered bacteria.” For sphingosine-1-phosphate (S1P), a more specific search combining “sphingosine-1-phosphate” or “S1P” with “IgA nephropathy” or “IgAN” was used to focus on evidence directly relevant to IgAN. English-language articles relevant to the clinical associations, biological mechanisms, and microbiota-targeted interventions discussed in this review were considered. Reference lists of relevant reviews and primary studies were also screened to identify additional pertinent publications. Priority was given to human studies directly related to IgAN, followed by experimental and mechanistic studies relevant to the proposed gut–kidney pathways. Because this article is a narrative review rather than a systematic review, no formal PRISMA-based study selection process or flow diagram was performed.
